# Acute median arcuate ligament syndrome after pancreaticoduodenectomy

**DOI:** 10.1186/s40792-016-0242-6

**Published:** 2016-10-15

**Authors:** Ilhan Karabicak, Sohei Satoi, Hiroaki Yanagimoto, Tomohisa Yamamoto, Satoshi Hirooka, So Yamaki, Hisashi Kosaka, Masaya Kotsuka, Kentoro Inoue, Yoichi Matsui, Masanori Kon

**Affiliations:** Department of Surgery, Kansai Medical University, 2-5-1, Shin-machi, Hirakata, Osaka 573-1010 Japan

**Keywords:** Median arcuate ligament syndrome, pancreaticoduodenectomy, acute onset

## Abstract

Median arcuate ligament syndrome (MALS) has been reported in 2–7.6 % of patients undergoing pancreaticoduodenectomy (PD). Most of the reported cases of MALS have been diagnosed perioperatively and treated radiologically or surgically before or during PD. MALS can have an acute postoperative onset after PD even if all preoperative and intraoperative evaluations are normal particularly in young patients.

In this report, we present a second case of severe hepatic cytolysis secondary to MALS that developed acutely and the first patient who required acute division of the median arcuate ligament after PD.

## Background

The incidence of celiac axis stenosis (CAS) caused mainly by median arcuate ligament syndrome (MALS) is reported around 7.3 % in asymptomatic individuals [1]. MALS has been reported in 2–7.6 % of patients undergoing pancreaticoduodenectomy (PD) [2–5].

The mechanism of MALS has been well demonstrated. The median arcuate ligament is a tendinous band spanning the right and left diaphragmatic crura, anterior to the aorta. In MALS, the celiac artery (CA) is compressed by this fibrous band, causing extrinsic compression anteriorly and leading to partial or complete CA occlusion [2, 6].

It is crucial to diagnose MALS in the preoperative period when planning PD. The pancreaticoduodenal arcades represent the largest collateral circle to allow retrograde flow through the gastroduodenal artery (GDA) when CAS occurs; this retrograde flow allows for blood to supply the liver, stomach, spleen, and pancreas [7]. Breakdown of this collateral systems during PD in a patient with MALS will cause ischemia of the liver and anastomotic failure [8, 9].

Most of the reported cases of MALS have been diagnosed perioperatively, either by radiologic findings or by intraoperative digital palpation or Doppler sonography [2, 8–10]. When MALS is diagnosed preoperatively, it can be treated by interventional radiology or intraoperatively by median arcuate ligament division or by pass grafting [8, 9, 11–16]. Even when diagnosed perioperatively, MALS can cause severe complications and even mortality after division of the median arcuate ligament or reconstruction of the CA [3, 11, 12, 17–20].

Acute onset MALS during or after PD is very rare. Unfortunately, acute MALS can develop after PD in patient without any radiologic signs of MALS. Sanchez et al. [7] reported the first patient diagnosed with MALS 1 day after PD in 2013. Although they were able to treat their patient conservatively, there is no standardized approach to this situation.

In this report, we present a patient with MALS that developed acutely after PD, caused severe hepatic cytolysis, and required acute division of the median arcuate ligament.

## Case presentation

A 40-year-old male presented to our hospital with a 2-month history of back pain. Physical examination showed no abnormalities, and laboratory examination was normal other than an elevated amylase level. Abdominal contrast-enhanced computed tomography (CT) revealed a low-density mass with a diameter of 20 mm at the pancreatic head, with segmental portal vein (PV) attachment. Endoscopic ultrasonography was performed, and a fine-needle biopsy sample showed adenocarcinoma of the pancreas. Diagnostic laparoscopy ruled out latent peritoneal or liver metastasis. The patient received neoadjuvant chemoradiotherapy (NACRT) using oral fluoropyrimidine (S-1) for 28 days. Post-NACRT CT showed no change in tumor size or in PV attachment.

The patient then underwent PD; intraoperative exploration showed normal anatomy of the celiac trunk, mesenteric vessels, and related branches. Clamp testing of the GDA showed normal hepatic artery pulsation. We performed PD with PV resection and reconstruction and extended lymphadenectomy, including lymph-node dissection around the celiac trunk. Before closing the abdomen, we noted that pulsation of the common hepatic artery became weak, but blood regurgitation was of adequate strength after reopening the cut stump of the right gastric artery. The duration of surgery was 486 min, and the blood loss was 747 mL; blood transfusion was not required. On postoperative day 1, the patient was in stable condition but his liver function tests were abnormal. The preoperative liver enzymes, international normalized ratio (INR), complete blood-cell count (CBC), and tumor markers had all been normal. However, within 12 h of PD, the liver enzymes, INR, white blood cell (WBC) count, platelet (PLT) count, and C-reactive protein (CRP) were abnormal and rapidly worsened over the next 12 h (Table 1).

**Table 1:** The preoperative and postoperative abnormal laboratory values

	Preoperative	PO 2nd hour	PO 12th hour	PO 20th hour	Post re-op, 12th hour	Post re-op 4th day
AST (U/L)	25	761	2090	2656	1824	64
ALT (U/L)	27	866	2101	2663	2165	458
INR	0.98	1.36	1.72	1.99	1.27	1.06
WBC (uL)	39	56	105	105	87	54
PLT (uL)	14.8	9.7	11.6	12.2	8.3	12
CRP (mg/dL)	0.025	0.398	6.106	10.424	12.871	2.339

*Abbreviations*: *PO* postoperative, *AST* aspartate aminotransferase, *ALT* alanine aminotransferase, *INR* international normalized ratio, *WBC* white blood cell, *PLT* platelet, *CRP* C-reactive protein

**Fig. 1:**
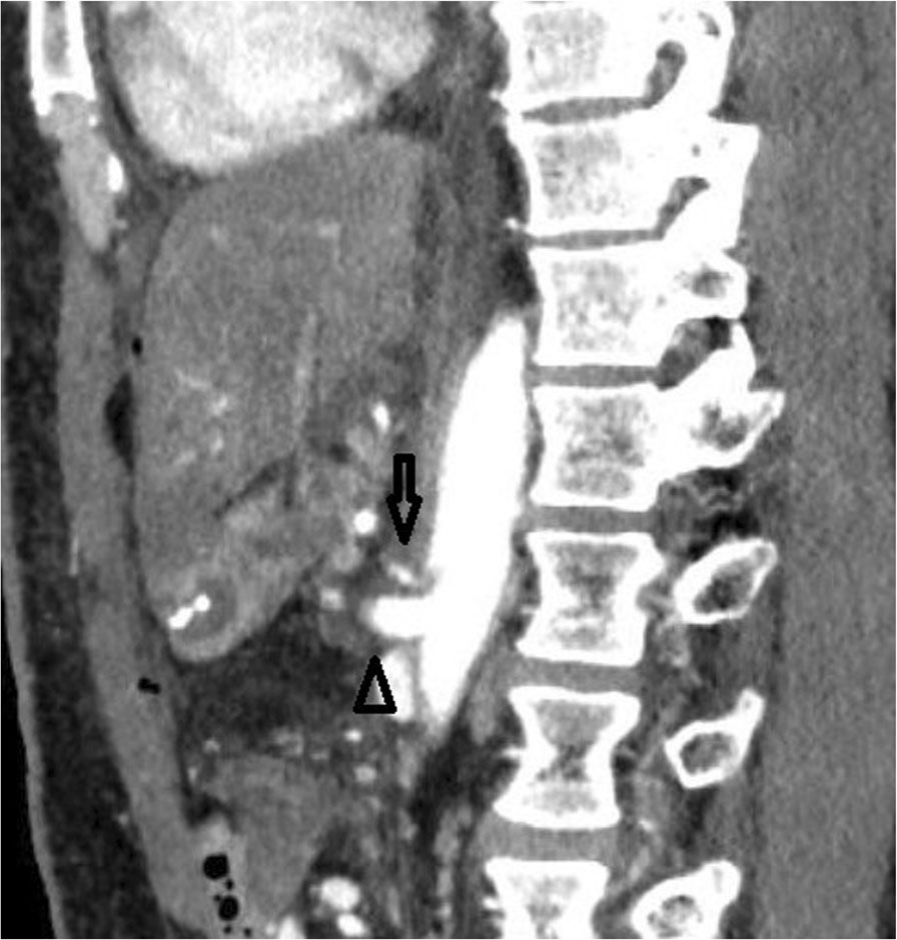
Preoperative multidetector CT with routine arterial reconstruction showing normal celiac artery anatomy and no evidence of median arcuate ligament syndrome (the *arrow* shows the CA and the *arrow head* shows the SMA)

**Fig. 2:**
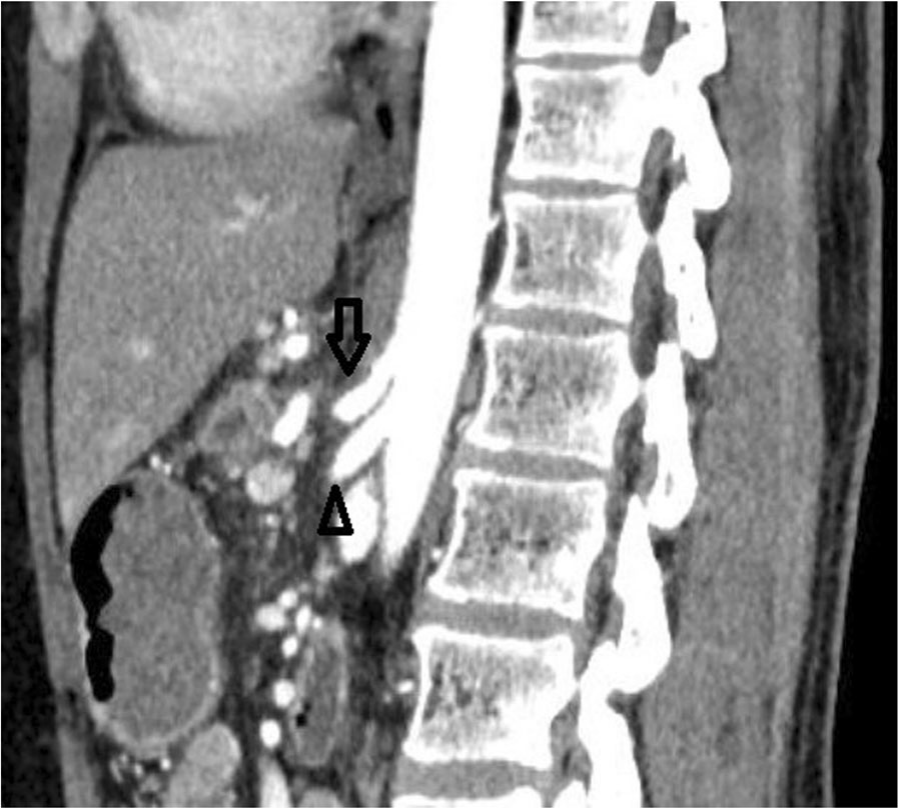
Multidetector CT with arterial reconstruction 1 day after the PD showing an acute extrinsic stenosis caused by median arcuate ligament compression

**Fig. 3:**
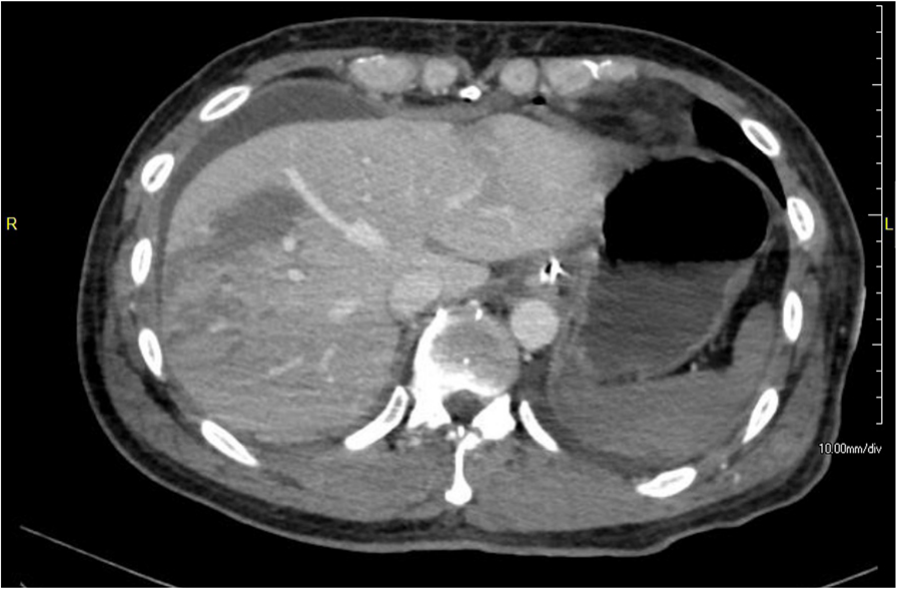
Multidetector CT 1 day after the PD showing widespread liver ischemia (the *arrow* shows the CA and the *arrow head* shows the SMA)

Although the preoperative CT with routine arterial reconstruction had shown normal CA anatomy and no evidence of MALS, a postoperative scan 1 day after the PD that included a lateral projection of the CA showed an acute extrinsic stenosis caused by newly developed compression caused by the median arcuate ligament. Widespread liver ischemia was also apparent (Figs. 1, 2, and 3).

The patient underwent urgent reoperation for acute onset MALS causing severe hepatic cytolysis. There was no palpable blood flow at the celiac trunk, and flow at the hepatic artery was markedly decreased. Bile leakage from the suture points of the hepaticojejunostomy was detected; this seemed to be caused by decreased blood flow in the common hepatic artery. The median arcuate ligament was released, with subsequent dramatic resumption of celiac trunk and common hepatic artery hepatic artery pulsation.

**Fig. 4:**
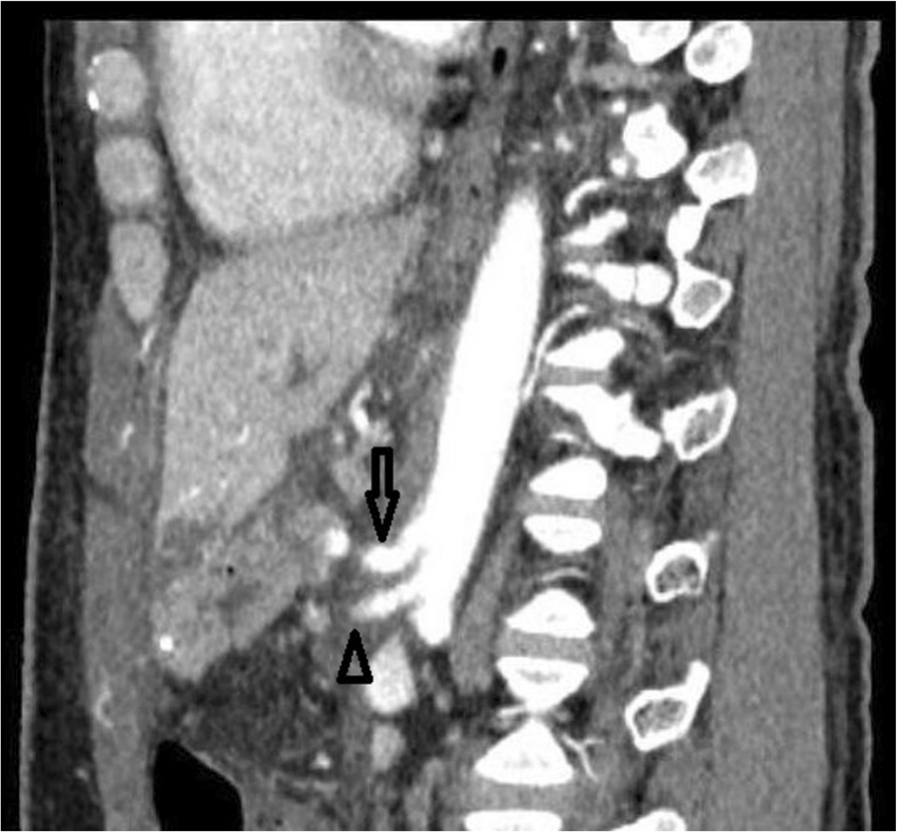
CT scan after the second operation showed that the stenosis of celiac axis was fully released (the *arrow* shows the CA and the *arrow head* shows the SMA)

**Fig. 5:**
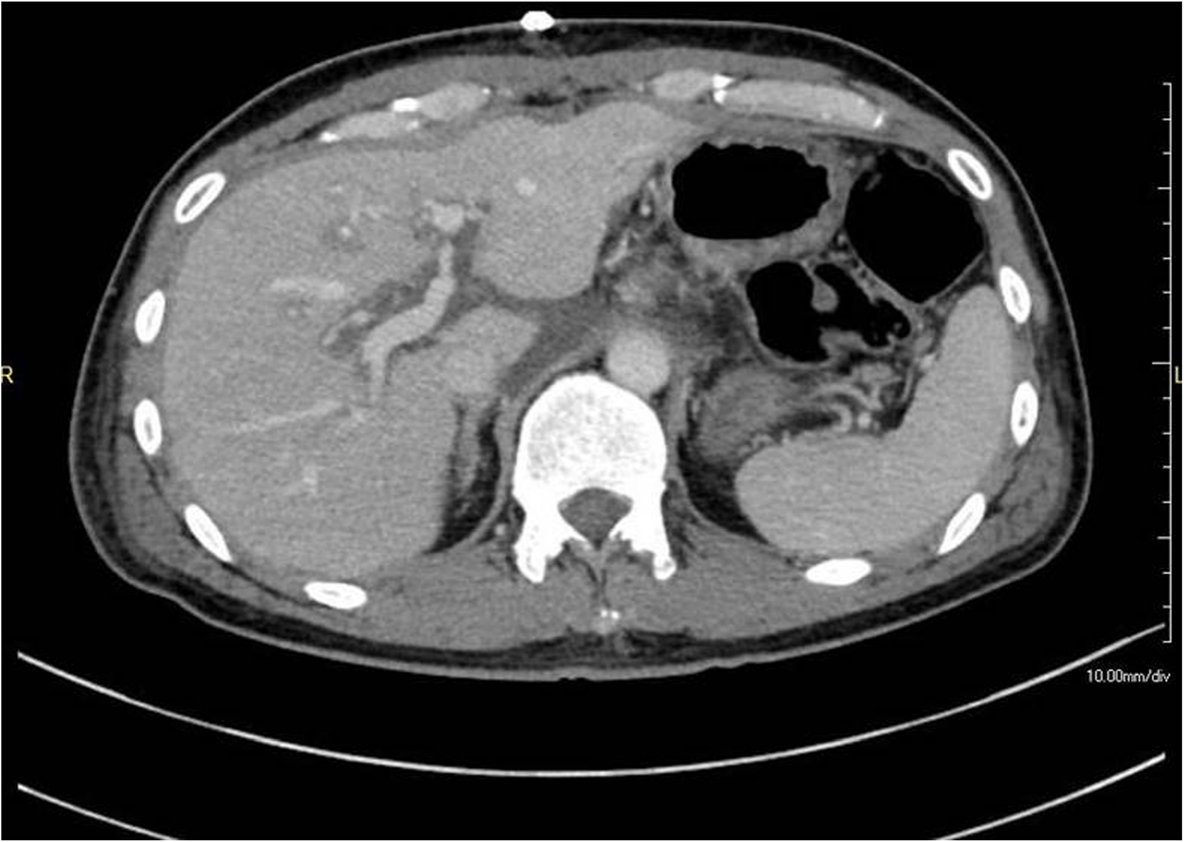
CT scan 11 days after reoperation showed that the area of liver ischemia was reduced

After division of the median arcuate ligament, the patient’s liver enzymes, INR, and WBC gradually normalized. Eleven days after reoperation, CT showed regular flow into the celiac trunk, the proper hepatic artery, and the PV; the area of liver ischemia was reduced (Figs. 4 and 5). The patient did not experience any regurgitant cholangitis and liver abscess. Prompt MALS release was succesful in order to prevent any major complications. The patient required a longer than usual postoperative stay in order to recover from bile leakage. He was eventually discharged 43 days after the original PD, in good condition.

### Discussion

The pancreaticoduodenal arcades make up the major collateral circle that allows retrograde flow through the GDA in case of CAS. They allow for blood to reach the hepatic, gastric, splenic, and pancreatic arteries when the celiac trunk is compromised [7].

The preoperative diagnosis of MALS is essential so that this arcade may be preserved [8, 9]. It is not always possible to diagnose MALS before proceeding with pancreatic resection during PD. Failure to demonsrate MALS before or during PD could potentially cause major morbidity, leading to ischemic and fatal complications [11, 17–19].

Three-dimensional CT angiography shows a characteristic hook pattern on the anterior proximal celiac axis when it is compressed by the median arcuate ligament [13, 14]. Gaujoux et al. [15] reported that multidetector CT, including lateral views, can detect significant arterial stenosis with 96 % sensitivity and determine the etiology of CA stenosis with 92 % accuracy.

If MALS is diagnosed before PD, various methods are available for revascularization before or during the procedure: these include open or laparoscopic median arcuate ligament division, a vascular bypass procedure, or endovascular stenting [16–18, 21–25].

Intraoperative trial clamping of the GDA should be performed before breakdown of the collateral circulation during PD, even if preoperative CT does not demonstrate MALS [2]. If MALS is present, this clamping will markedly decrease the hepatic artery pulsation as evaluated by digital palpation or intraoperative Doppler ultrasonography [3]. If MALS is diagnosed during PD, the median arcuate ligament must be divided at the beginning of the procedure, before GDA ligation or pancreatic division [15, 20]. This safe and fast procedure permits trunk decompression and resolution of ischemic disorders in up to 89 % of patients [15].

MALS can develop acutely after PD in patients with normal hepatic artery flow during GDA clamping. Sanchez et al. [7] reported the first patient with acute onset MALS after PD who did not demonstrate hepatic artery flow impairment during GDA clamping. They were able to treat their patient conservatively. We report herein a second such patient who had no evidence of hepatic artery flow impairment during GDA clamping and pancreatic transection.

Since the appearance of MALS after PD is very rare, the appropriate treatment has to be determined according to the patient’s general status. We decided on exploratory laparotomy since our patient had abundant hepatic cytolysis. Also influencing our decision, his liver enzymes, INR, and CRP worsened abruptly within 12 h of the original surgery and rapidly increased by 24 h after PD. Repeat CT also showed widespread liver ischemia. At re-exploration, we noted bile leakage from the suture points of the hepaticojejunostomy, probably due to the decreased blood flow from the common hepatic artery. Eventually, our patient developed a biliary fistula that required extended hospitalization.

There is no known explanation for this acute onset of MALS after PD in a patient with normal celiac anatomy. Sanchez et al. [7] tried to explain this phenomenon as occurring in patients with pre-existing nonsignificant CAS that is exacerbated by either extended lymphadenectomy of the celiac region or by the prolonged bent back position of the patient during surgery. However, our patient’s celiac lymphadenectomy and duration of the surgery were not different from our other patients who have not developed MALS. Although our patient underwent NACRT followed by PD, the field of radiation did not include the region of the celiac trunk. We hypothesize that a very tight median arcuate ligament presented in this young patient in addition to lymph-node clearance around the celiac trunk might induce the stenosis.

## Conclusions

MALS is most often diagnosed preoperatively, and a small portion of patients are diagnosed intraoperatively. Unfortunately as in our patient, MALS can have an acute postoperative onset even if all preoperative and intraoperative evaluations are normal. Hepatic cytolysis after PD with extended lymphadenectomy should alert clinicians to the possibility of MALS, even in patients with normal radiological vascular anatomy. Patients who acutely deteriorate despite medical treatment should undergo surgical exploration to rule out other conditions and division of the median arcuate ligament in order to prevent any major complications if MALS is indeed present.

## Abbreviations

CAS: Celiac axis stenosis
CBC: Complete blood-cell count
CRP: C-reactive protein
CT: Computed tomography
GDA: Gastroduodenal artery
INR: International normalized ratio
MALS: Median arcuate ligament syndrome
NACRT: Neoadjuvant chemoradiotherapy
PD: Pancreaticoduodenectomy
PLT: Platelet
PV: Portal vein
WBC: White blood cell
